# Research on green decision making of pharmaceutical logistics considering government subsidy strategy

**DOI:** 10.1371/journal.pone.0241400

**Published:** 2020-10-30

**Authors:** Zhe Huang, Min Fu

**Affiliations:** School of Business Administration, Shenyang Pharmaceutical University, Shenyang, Liaoning, China; Institute for Advanced Sustainability Studies, GERMANY

## Abstract

In view of the difficulty, high cost and complex technology of pharmaceutical logistics green transformation, based on the idea of green supply chain, three different government subsidy strategies for green logistics were proposed. Firstly, by constructing a Stackelberg game model with pharmaceutical logistics provider as the leader and manufacturer as the followers, the behavior selection and optimal decisions of the participants under the anarchic subsidy strategy, the single subsidy strategy of the pharmaceutical logistics provider, the single subsidy strategy of the pharmaceutical manufacturer and the coordinated subsidy strategy are analyzed respectively. Furthermore, the effects of different subsidy strategies on the green investment and strategy selection of logistics provider and manufacturer are compared. Finally, according to the research results, the paper provides reference and suggestions for the formulation of government subsidy strategy. The results show that the three subsidy strategies have different degrees of incentive effect on the green transformation of pharmaceutical logistics, and the single logistics provider subsidy strategy is the best.

## Introduction

Pharmaceutical green logistics has been defined as the logistics activities that maximize the utilization of logistics resources and minimize the adverse impact of logistics activities on the environment in the process of pharmaceutical products logistics, so as to realize environmental evolution [1]. Due to the particularity of pharmaceutical products, the current attention to their logistics activities is mostly focused on safety and timeliness, while the attention to the green development of pharmaceutical logistics is relatively low. However, with the rapid development of pharmaceutical logistics industry, the consumption and carbon emissions of pharmaceutical products in transportation and packaging are increasing, which has brought great pressure to the environment. Pharmaceutical products can not only by providing material foundation to make great contributions to human health, implement pharmaceutical products circulation greening is the important measure of protecting people's health [2], so the medicine logistics must be pay attention to energy conservation and emission reduction, the implementation of the green logistics from start to green development, promote the sustainable development of the industry. However, the cost problem brought by the greening of logistics has largely hindered its development, which is more obvious in the pharmaceutical logistics industry. As medical logistics is more professional, with higher safety and technical requirement, the cost and difficulty of green transformation are greater. Therefore, in order to encourage the green logistics behavior of medical logistics provider and manufacturers, it is extremely important to provide appropriate policy subsidies [3]. On this basis, from the perspective of government subsidies, considering the impact of different subsidy strategies on the green transformation of medical logistics is a research topic of theoretical value and practical significance.

In recent years, the green subsidy of pharmaceutical logistics is rarely studied. However, some research results on pharmaceutical logistics and government green subsidy policy are shown. In terms of pharmaceutical logistics, Chakib Bojji et al. comprehensively analyzed the research on logistics in the existing pharmaceutical supply chain, discussed different coordination modes of pharmaceutical supply chain, clarified the different obstacles in the management of pharmaceutical supply chain, and pointed out the possible research directions in the future [4]. Fu Haoran et al. used case analysis method to analyze the development status of Deppon Green logistics and pointed out the problems existing in the construction process of deppon green logistics system. From the perspective of enterprises, it proposes effective ways to promote the development of green logistics [5]. Shen Jianfang analyzed the key problems existing in the current development of domestic pharmaceutical logistics, sorted out the requirements of the transformation and development of pharmaceutical logistics on logistics mode innovation and standardization implementation, and put forward relevant development countermeasures [6]. Tang Jianrong et al. used empirical analysis method to analyze the change of green TFP in China's logistics industry, and put forward Suggestions for promoting green development of logistics [7]. Zhang Tiantian studied the current situation of green development of pharmaceutical logistics in Anhui Province of China, and pointed out the problems in the process of development, such as incomplete facilities and low degree of specialization [8]. Wang Cuimin et al. studied the relevant countermeasures to promote the development of green pharmaceutical logistics industry in Hebei Province of China under the new normal [9]. In terms of government green subsidy strategies, Yuan Xigang et al. studied different government subsidy strategies in green supply chain management, and the research results showed that government subsidy strategies could not only effectively improve the greenness of products in the green supply chain, but also increase the profits of enterprises and help retailers to improve their sales efforts [10]. Mitra S et al. established a two-stage game model between producers and remanufacturers, and analyzed the important role of government subsidies in green remanufacturing activities by comparing the government subsidies to remanufacturers, producers, remanufacturers and producers at the same time [11]. WANG et al. studied manufacturers' subsidy strategies and assumed that subsidy credits were related to the cost of introducing low-carbon technologies, the cost of R&D and innovation of green products, and the cost of recovery and reprocessing of remanufactured products, and found that government subsidies would encourage manufacturers to improve their green efforts [12]. Jiang Shiying et al. established a three-stage game model with the government, the manufacturer and the manufacturer as the main body, took the two-level green supply chain composed of green manufacturers and manufacturers as the research object, and analyzed the effect of government subsidies by numerical simulation [13]. Based on the idea of green supply chain, Zhu Qinghua et al. introduced the concept of product greenness, established a three-stage game model for each participant under the condition of government subsidies, explored the impact of various parameter changes, and provided support for the government and supply chain manufacturers to make green supply chain management decisions [14].

The existing studies on pharmaceutical logistics provide us with the development status of the pharmaceutical logistics industry in China, and sort out the operation mode, technical characteristics and development trend of the industry. At the same time, the existing problems such as low degree of intelligence and difficulty in green transformation are pointed out. All of these contribute to our acquisition of relevant basic knowledge and understanding of the real background [15–18]. The research on government green subsidy strategy provides necessary technical support for innovation of subsidy strategy, establishment of game model and selection of calculation method [19–23]. However, the existing research has not put forward the subsidy strategy with guiding significance for the green development of medical logistics, and the research on green medical logistics is relatively deficient. It should be pointed out that, with the development of green sharing economy, green logistics is bound to become a trend, which is more important in the field of medicine with higher professionalism. Therefore, different from previous studies, this paper constructs three different subsidy strategies for green pharmaceutical logistics from the perspective of green supply chain, and uses comparative analysis to explore the impact of different government subsidies on the greening of pharmaceutical logistics, so as to provide reference for the behavior decision-making of pharmaceutical green logistics participants. First, the development status of green medicine logistics under the anarchic subsidy strategy is analyzed, and the basic profits and contribution of green logistics level of each participant are calculated. On this basis, we explore the subsidy strategy to improve the overall utility. Then, based on the idea of green supply chain, green packaging and green transportation are mainly considered. Different strategies of subsidies are implemented for logistics provider and manufacturer in the supply chain, and their respective profit parameters are analyzed and calculated. Through further comparative analysis, the influence of the change of strategy on the optimal profit and green efforts of each participant is studied. On this basis, the most appropriate government subsidy strategy is selected to promote the green packaging and transportation, so as to promote the development of green logistics in the pharmaceutical industry.

The remaining sections of the paper are organized as follows. Section 2 introduces the relevant assumptions of the model, and explains the meaning of the relevant symbols, which lays the foundation for the construction and analysis of the model. Section 3 constructs the basic model and game model under different subsidy policies, and analyzes the behavior decision of pharmaceutical logistics provider and manufacturer under each model. Section 4 Finally, the conclusions of this paper with respect to its original contributions and suggested future work are discussed.

## Preliminaries

In this section, the problem description and research hypothesis are introduced below.

### Problem description

Due to the particularity of medical products and the high cost of medical logistics, the public mostly pay attention to the safety and cost reduction of medical logistics, so they often ignore the environmental protection of logistics. However, there are a lot of waste of resources and environmental damage in the existing medical logistics, such as repeated packaging, excessive logistics circulation, and exhaust emission of transportation vehicles [24]. On this basis, a secondary green supply chain consisting of a pharmaceutical logistics provider and a pharmaceutical manufacturer is taken as the research object in this paper. The government formulates several different subsidy strategies for green logistics, pharmaceutical logistics provider and pharmaceutical manufacturer make corresponding logistics behavior choices and logistics decisions according to the costs and benefits of green logistics. Pharmaceutical logistics provider can transform green logistics by using green transportation vehicles and green packaging. Retailers have a green logistics preference and are willing to pay extra for pharmaceutical products shipped in a green logistics mode. Pharmaceutical manufacturer mainly contributes to the transformation of green logistics through green packaging and green publicity in the sales process. This paper intends to solve the following three questions as follows:

1. How do different subsidy strategies influence the choice of logistics behavior of pharmaceutical logistics provider and manufacturer?
2. In the case of a certain amount of government subsidies, what are the different effects of different subsidy strategies?
3. Compared with the non subsidy situation, does the government's different subsidy strategies significantly promote the green process of pharmaceutical logistics?

### Research hypothesis

Based on the above research questions, the following assumptions were made for the model:

1. This paper only studies the pharmaceutical logistics behavior, considering only the Stackelberg game situation in which the logistics provider is the leader and the manufacturer is the follower. In this supply chain, logistics provider undertakes the task of logistics transportation and collect the corresponding logistics fees. Manufacturers sell products to retailers, and the logistics cost is added to the product price.
2. As green logistics is dependent on products, its costs and benefits are finally reflected in the products, and also affect the greenness and price of medical products. Medical products that use green logistics are slightly more expensive.
3. The promotion of green logistics is related to the use of logistics price, green level and the green investment of pharmaceutical logistics provider and manufacturer. Retailers are willing to pay extra price for the use of green logistics for medical products. The market demand of green pharmaceutical logistics depends on medical products, so we can describe the market demand of "green logistics" as follows:
  d=α‐βp+γz (1)
  Where, *d* denotes the demand of green logistics; *α* denotes the overall size of the logistics market, *α*−*βp*>0;*β* denotes the price sensitivity coefficient of logistics demand; *p* denotes the logistics fee charged by the logistics provider to the retailer; *γ* denotes the retailer's green logistics preference coefficient; *e* denotes the green level of logistics contributed by the logistics provider; *z* represents the green level of logistics contributed by manufacturers [25].
4. When realizing green transformation of logistics, pharmaceutical logistics provider and manufacturer do not change the original logistics unit cost, but need to increase additional green investment (equipment transformation and technology innovation) I. Assuming a quadratic relation between green input cost and greenness, then let *I*_*L*_ = *η*_*L*_*e*^2^/2,*I*_*R*_ = *η*_*R*_*z*^2^/2, Where, η denotes the coefficient of green input cost [26].
5. Assume that all participants of the green supply chain are completely rational and make corresponding behavioral choices and decisions according to their own interests maximization.

In order to effectively encourage the green logistics transformation of pharmaceutical logistics provider and manufacturers, the government considers various subsidy strategies and studies the influence of different subsidy strategies on the green decision-making of each participant in the supply chain. This paper mainly considers the following situations: anarchic subsidy strategy, the single subsidy strategy of pharmaceutical logistics provider, the single subsidy strategy of pharmaceutical manufacturer and the coordination subsidy strategy. On this basis, a Stackelberg game model with pharmaceutical logistics provider as the leader and manufacturer as the follower is built to explore the behavior choice and optimal decision of each participant under different subsidy strategies. First of all, the *s*, where *s* denotes the subsidy coefficient of green logistics is determined by the government. And then the green input and unit price of green logistics are determined by the pharmaceutical logistics provider according to government subsidies, so as to optimize their own profits. According to the unit price and the green level of logistics determined by the logistics provider, the charge of logistics fees and its own logistics green investment (mainly through green packaging and green publicity) are determined by the pharmaceutical manufacturer, so as to optimize its own profit. In order to simplify the description, the anarchic subsidy strategy, the logistics subsidy strategy, the manufacturer subsidy strategy and the coordination subsidy strategy are represented by the subscripts n, l, r and x.

## Model construction and analysis

The problem description and research hypothesis are first introduced, then the four different subsidy strategy are presented.

### Anarchy subsidy strategy

Under the anarchic subsidy strategy, the government does not conduct subsidy in different forms. Under this strategy, pharmaceutical logistics provider and manufacturer invest green costs independently to improve the green level of logistics.

The profit function of pharmaceutical logistics provider (L) is as follows:

$$\pi_{n}^{L}=(v-c)(\alpha -\beta p+\gamma e_{n}+\gamma z_{n})-\eta_{L}e_{n}^{2}/2$$ (2)

The profit function of pharmaceutical manufacturer (R) is as follows:

$$\pi_{n}^{R}=(p-v)(\alpha -\beta p+\gamma e_{n}+\gamma z_{n})-\eta_{R}z_{n}^{2}/2$$ (3)

Total profit function of green supply chain (B) function is as follows:

$$\pi_{n}^{B}=\pi_{n}^{L}+\pi_{n}^{R}=(p-c)(\alpha -\beta p+\gamma e_{n}+\gamma z_{n})-\eta_{L}e_{n}^{2}/2-\eta_{R}z_{n}^{2}/2$$ (4)

The decision order is determined after the model is established. This paper constructs a single supply chain consisting of pharmaceutical logistics provider and manufacturer, and only considers their behavior selection and decision-making. First of all, pharmaceutical logistics provider, as leader, should first set logistics carrier price v and contribution e_n_ of logistics green level to provide corresponding logistics services to manufacturer. Then, as a follower, the manufacturer decides its logistics cost plus price and green investment effort level according to the carrier price and green degree of the logistics provider, so that the pharmaceutical products can be delivered to the retailer through green logistics. Based on the above decision order, this paper use backward induction to solve:

$$\{\begin{array}{c} \alpha -2\beta p+\beta v+\gamma e_{n}+\gamma z_{n}=0\\ \gamma p-\gamma v-z_{n}\eta_{R}=0 \end{array}$$ (5)

The above simultaneous equations can be solved as follows:

$$\{\begin{array}{c} p=\frac{\alpha \eta_{R}-\gamma^{2}v+\beta v\eta_{R}+\gamma e_{n}z_{n}}{\gamma^{2}-2\beta \eta_{R}}\\ z_{n}=\frac{\beta v\gamma -\alpha \gamma -\gamma^{2}e_{n}}{\gamma^{2}-2\beta \eta_{R}} \end{array}$$ (6)

When the manufacturer's green logistics plus price p and the green logistics level *z*_*n*_ contributed by the manufacturer are both greater than 0, the extreme value is taken here. Then, p and *z*_*n*_ in (6) are substituted into Eq (2) to find the partial derivatives of v and *e*_*n*_ respectively. The simultaneous equations can be solved as follows:

$$\{\begin{array}{c} v^{*}=\frac{-c\beta \gamma^{2}\eta_{R}+(\alpha +c\beta )\eta_{L}(2\beta \eta_{R}-\gamma^{2})}{-2\beta \eta_{L}(\gamma^{2}-2\beta \eta_{R})-\beta \gamma^{2}\eta_{R}}\\ e_{n}^{*}=\frac{\gamma \eta_{R}(\alpha -c\beta )}{-2\eta_{L}(\gamma^{2}-2\beta \eta_{R})-\gamma^{2}\eta_{R}} \end{array}$$ (7)

In this paper, the hessian matrix is used to verify the solving conditions of the equilibrium solution. According to hessian matrix, in order to obtain the above equilibrium solution, *β*^2^*η*_*R*_[*γ*^2^*η*_*R*_+2*η*_*L*_(*γ*^2^−2*βη*_*R*_)]≻0 is needed. Then, the above equilibrium solution v* and *e*_n_* are substituted into Eq (7) to obtain the optimal solution:

$$\{\begin{array}{c} p^{*}=\frac{-c\beta \gamma^{2}\eta_{R}+\eta_{L}\left[-\left(\alpha +c\beta \right)\gamma^{2}+\beta \left(3\alpha +c\beta \right)\eta_{R}\right]}{-2\beta \eta_{L}\left(\gamma^{2}-2\beta \eta_{R}\right)-\beta \gamma^{2}\eta_{R}}\\ z_{n}^{*}=\frac{\gamma \eta_{L}\left(c\beta -\alpha \right)}{2\eta_{L}\left(\gamma^{2}-2\beta \eta_{R}\right)+\gamma^{2}\eta_{R}} \end{array}$$ (8)

According to the actual problem, Eq (8) and Eq (7) can be substituted into Eq (3) and Eq (2) respectively to obtain the optimal profit of the medical logistics provider and manufacturer:

$$\{\begin{array}{c} \pi_{L}^{*}=\frac{-{\left(\alpha -c\beta \right)}^{2}\eta_{L}\eta_{R}}{2\gamma^{2}\eta_{R}+4\eta_{L}(\gamma^{2}-2\beta \eta_{R})}\\ \pi_{R}^{*}=\frac{{\left(\alpha -c\beta \right)}^{2}\eta_{L}^{2}\eta_{R}(2\beta \eta_{R}-\gamma^{2})}{2{\left[\gamma^{2}\eta_{R}+2\eta_{L}(\gamma^{2}-2\beta \eta_{R})\right]}^{2}} \end{array}$$ (9)

At this point, the optimal profit of the supply chain is as follows:

$$\pi_{B}^{*}=\pi_{L}^{*}+\pi_{R}^{*}$$ (10)

### Single subsidy strategy for pharmaceutical logistics provider

Due to the high requirements of the pharmaceutical industry for the safety, timeliness and professionalism of logistics, and the strict cold chain transport to vaccines, blood products and other products, the difficulty and cost of green transformation of pharmaceutical logistics is greater than that of general commodity logistics. Pharmaceutical logistics provider plays an important role in the green transformation of medical logistics, but the high cost investment reduces the enthusiasm. Therefore, in order to encourage the green transformation of pharmaceutical logistics companies, the government carries out a single subsidy strategy. The subsidy amount is affected by the green level *e*_*L*_ contributed by the logistics provider. Assume that the subsidy coefficient of the government is s(1≥ s≥0). On this basis, under the single subsidy mode of pharmaceutical logistics provider, the profit functions of logistics provider and manufacturer are as follows:

$$\pi_{l}^{L}=(v_{l}-c)(\alpha -\beta p_{l}+\gamma e_{l}+\gamma z_{l})-\eta_{L}e_{l}^{2}/2+s\eta_{L}e_{n}^{2}/2$$ (11)

$$\pi_{l}^{R}=(p_{l}-v_{l})(\alpha -\beta p_{l}+\gamma e_{l}+\gamma z_{l})-\eta_{R}z_{l}^{2}/2$$ (12)

The total profit of green supply chain is as follows:

$$\pi_{l}^{B}=(p_{l}-c)(\alpha -\beta p_{l}+\gamma e_{l}+\gamma z_{l})-\eta_{L}e_{l}^{2}/2-\eta_{R}z_{l}^{2}/2+s\eta_{L}e_{l}^{2}/2$$ (13)

Similarly, the inverse induction method is used to verify the existence conditions of the equilibrium solution according to hessian matrix. When 2*βη*_*R*_−*γ*^2^≻0 and *β*^2^*η*_*R*_[*γ*^2^*η*_*R*_−2(*s*−1)*η*_*L*_(*γ*^2^−2*βη*_*R*_)]≺0, the following equation is obtained:

$$\{\begin{array}{c} v_{l}^{*}=\frac{c\beta \gamma^{2}\eta_{R}+(\alpha +c\beta )(s-1)\eta_{L}(2\beta \eta_{R}-\gamma^{2})}{\beta \left[\gamma^{2}\eta_{R}-2(s-1)\eta_{L}(\gamma^{2}-2\beta \eta_{R})\right]}\\ e_{l}^{*}=\frac{\gamma \eta_{R}(c\beta -\alpha )}{\gamma^{2}\eta_{R}-2(s-1)\eta_{L}(\gamma^{2}-2\beta \eta_{R})} \end{array}$$ (14)

$$\{\begin{array}{c} p_{l}^{*}=\frac{c\beta \gamma^{2}\eta_{R}-(s-1)\eta_{L}\left[\left(\alpha +c\beta \right)\gamma^{2}-\beta (3\alpha +c\beta )\eta_{R}\right]}{\beta \left[\gamma^{2}\eta_{R}-2(s-1)\eta_{L}(\gamma^{2}-2\beta \eta_{R})\right]}\\ z_{l}^{*}=\frac{(\alpha -c\beta )\gamma (s-1)\eta_{L}}{\gamma^{2}\eta_{R}-2(s-1)\eta_{L}(\gamma^{2}-2\beta \eta_{R})} \end{array}$$ (15)

By substituting Eqs (14) and (15) into Eqs (11) and (12) respectively, the optimal profits of pharmaceutical logistics provider and manufacturer can be obtained as follows:

$$\{\begin{array}{c} \pi_{l}^{L*}=\frac{{\left(\alpha -c\beta \right)}^{2}(s-1)\eta_{R}\eta_{L}}{2\gamma^{2}\eta_{R}-4(s-1)\eta_{L}(\gamma^{2}-2\beta \eta_{R})}\\ \pi_{l}^{R*}=\frac{{\left(\alpha -c\beta \right)}^{2}{\left(s-1\right)}^{2}\eta_{L}{}^{2}\eta_{R}(2\beta \eta_{R}-\gamma^{2})}{2{\left[\gamma^{2}\eta_{R}-2(s-1)\eta_{L}(\gamma^{2}-2\beta \eta_{R})\right]}^{2}} \end{array}$$ (16)

At this time, the optimal profit of the supply chain is as follows:

$$\pi_{l}^{B*}=\pi_{l}^{L*}+\pi_{l}^{R*}$$ (17)

Proposition 1: By comparing and analyzing the changes of green level contribution, optimal profit and overall benefit of supply chain of pharmaceutical logistics provider under single subsidy strategy and anarchy subsidy strategy, the results are as follows:

$e_{l}^{*}-e_{n}^{*}≻0、z_{l}^{*}-z_{n}^{*}≻0、\pi_{l}^{L*}-\pi_{L}^{*}≻0、\pi_{l}^{B*}-\pi_{n}^{B*}≻0.$ Proof: Because $e_{l}^{*}≻0,e_{n}^{*}≻0,\gamma^{2}\eta_{R}-2(s-1)\eta_{L}(\gamma^{2}-2\beta \eta_{R})-2\eta_{L}(\gamma^{2}-2\beta \eta_{R})-\gamma^{2}\eta_{R}=2s\eta_{L}(2\beta \eta_{R}-\gamma^{2})≺0,$ so $e_{l}^{*}-e_{n}^{*}≻0$, In the same way but card $z_{l}^{*}-z_{n}^{*}≻0$.

$\pi_{l}^{L*}-\pi_{L}^{*}=\frac{{\left(\alpha -c\beta \right)}^{2}(s-1)\eta_{R}\eta_{L}}{2\gamma^{2}\eta_{R}-4(s-1)\eta_{L}(\gamma^{2}-2\beta \eta_{R})}-\frac{-{\left(\alpha -c\beta \right)}^{2}\eta_{R}\eta_{L}}{2\gamma^{2}\eta_{R}+4\eta_{L}(\gamma^{2}-2\beta \eta_{R})}=\frac{s{\left(\alpha -c\beta \right)}^{2}\eta_{R}\eta_{L}-{\left(\alpha -c\beta \right)}^{2}\eta_{R}\eta_{L}}{2\gamma^{2}\eta_{R}+4\eta_{L}(\gamma^{2}-2\beta \eta_{R})-4s\eta_{L}(\gamma^{2}-2\beta \eta_{R})}-\frac{-{\left(\alpha -c\beta \right)}^{2}\eta_{R}\eta_{L}}{2\gamma^{2}\eta_{R}+4\eta_{L}(\gamma^{2}-2\beta \eta_{R})},$ Because $2\gamma^{2}\eta_{R}+4\eta_{L}(\gamma^{2}-2\beta \eta_{R})-4s\eta_{L}(\gamma^{2}-2\beta \eta_{R})≺2\gamma^{2}\eta_{R}+4\eta_{L}(\gamma^{2}-2\beta \eta_{R}),s{\left(\alpha -c\beta \right)}^{2}\eta_{L}\eta_{R}-{\left(\alpha -c\beta \right)}^{2}\eta_{L}\eta_{R}≻-{\left(\alpha -c\beta \right)}^{2}\eta_{L}\eta_{R},$ so $\pi_{l}^{L*}-\pi_{L}^{*}≻0$. In the same way but card $\pi_{l}^{R*}-\pi_{R}^{*}≻0$, $\pi_{l}^{B*}-\pi_{n}^{B*}=(\pi_{l}^{L*}+\pi_{l}^{R*})-(\pi_{L}^{*}+\pi_{R}^{*})≻0$.

Result analysis of proposition 1: When the government gives green subsidies to pharmaceutical logistics provider with subsidy coefficient s, the contribution of green level of pharmaceutical logistics provider and manufacturer is greater than that without subsidy, and the optimal profit of pharmaceutical logistics provider and manufacturer is greater than that under the anarchy subsidy strategy. In the meantime, profit of whole supply chain also increases accordingly. The results show that the implementation of single subsidy strategy by the government for pharmaceutical logistics provider can improve the self-interest and social welfare of each participant in the green supply chain, and encourage each participant to increase their contribution to the green level of logistics. Compared with the anarchy subsidy strategy, it has a more obvious promoting effect on the motivation of each participant to implement green behaviors and the improvement of the green level of logistics, and the overall effect of the strategy is better. On this basis, it is easier for pharmaceutical logistics company and manufacturer to accept this subsidy strategy.

### Single subsidy strategy for pharmaceutical manufacturer

In reality, most of the attention on the green transformation effect of pharmaceutical logistics is focused on logistics provider, and manufacturers' efforts are often ignored. However, as logistics runs through the whole process of pharmaceutical products circulation, manufacturer can also contribute to the development of green logistics by optimizing packaging and promoting green products. Especially in the pharmaceutical industry, consumers almost focus on the efficacy and safety of the product, ignoring the greenness of the product. Therefore, there are a lot of excessive packaging and waste of resources in the pharmaceutical market, which can be fundamentally changed through the green consciousness and green behavior of manufacturers. Therefore, the government should start from the reality, focus on the overall situation, pay attention to the contribution of pharmaceutical manufacturer in the process of logistics greening. In order to further stimulate the efforts of pharmaceutical manufacturer in green logistics, the government implemented a single subsidy strategy for them. It is assumed that the government subsidy amount is related to the logistics green level z contributed by the manufacturer, and the subsidy coefficient is s (1≥ s ≥0). On this basis, the profit functions of pharmaceutical logistics provider and manufacturer are established, which are as follows:

$$\pi_{r}^{L}=(v_{r}-c)(\alpha -\beta p_{r}+\gamma e_{r}+\gamma z_{r})-\eta_{L}e_{r}^{2}/2$$ (18)

$$\pi_{r}^{R}=(p_{r}-v_{r})(\alpha -\beta p_{r}+\gamma e_{r}+\gamma z_{r})-\eta_{R}z_{r}^{2}/2+s\eta_{R}z_{r}^{2}/2$$ (19)

The total profit of green supply chain is as follows:

$$\pi_{r}^{B}=\pi_{r}^{L}+\pi_{r}^{R}=(p_{r}-c)(\alpha -\beta p_{r}+\gamma e_{r}+\gamma z_{r})-\eta_{L}e_{r}^{2}/2-\eta_{R}z_{r}^{2}/2+s\eta_{R}z_{r}^{2}/2$$ (20)

Similarly, using the inverse induction, under the condition *β*^2^(*s*−1)*η*_*R*_{−*γ*^2^(*s*−1)*η*_*R*_+2*η*_*L*_[*γ*^2^+2*β*(*s*−1)*η*_*R*_]}≻0,−*γ*^2^−2*β*(*s*−1)*η*_*R*_≻0:

$$\{\begin{array}{c} v_{r}^{*}=\frac{-c\beta \gamma^{2}(s-1)\eta_{R}+(\alpha +c\beta )\eta_{L}\left[\gamma^{2}+2\beta (s-1)\eta_{R}\right]}{\beta \left\{-\gamma^{{}^{2}}(s-1)\eta_{R}+2\eta_{L}\left[\gamma^{2}+2\beta (s-1)\eta_{R}\right]\right\}}\\ e_{r}^{*}=\frac{(\alpha -c\beta )\gamma (s-1)\eta_{R}}{-\gamma^{{}^{2}}(s-1)\eta_{R}+2\eta_{L}\left[\gamma^{2}+2\beta (s-1)\eta_{R}\right]} \end{array}$$ (21)

$$\{\begin{array}{c} p_{r}^{*}=\frac{-c\beta \gamma^{2}(s-1)\eta_{R}+\eta_{L}\left[(\alpha +c\beta )\gamma^{2}+\beta (3\alpha +c\beta )(s-1)\eta_{R}\right]}{\beta \left\{-\gamma^{{}^{2}}(s-1)\eta_{R}+2\eta_{L}\left[\gamma^{2}+2\beta (s-1)\eta_{R}\right]\right\}}\\ z_{r}^{*}=\frac{(c\beta -\alpha )\gamma \eta_{L}}{-\gamma^{{}^{2}}(s-1)\eta_{R}+2\eta_{L}\left[\gamma^{2}+2\beta (s-1)\eta_{R}\right]} \end{array}$$ (22)

By substituting Eq (21) and Eq (22) into Eq (18) and Eq (19), the optimal profits of pharmaceutical logistics provider and manufacturer can be obtained as follows:

$$\{\begin{array}{c} \pi_{r}^{L*}=\frac{{\left(\alpha -c\beta \right)}^{2}\gamma^{2}\eta_{L}^{2}\eta_{R}}{{\left\{\gamma^{2}(s-1)\eta_{R}+2\eta_{L}\left[\gamma^{2}+2\beta (s-1)\eta_{R}\right]\right\}}^{2}}\\ \pi_{r}^{R*}=\frac{{\left(\alpha -c\beta \right)}^{2}(s-1)\eta_{L}^{2}\eta_{R}\left[\gamma^{2}+2\beta (s-1)\eta_{R}\right]}{2{\left\{\gamma^{2}(s-1)\eta_{R}+2\eta_{L}\left[\gamma^{2}+2\beta (s-1)\eta_{R}\right]\right\}}^{2}} \end{array}$$ (23)

At this time, the optimal profit of the supply chain is as follows:

$$\pi_{r}^{R*}=\pi_{r}^{L*}+\pi_{r}^{R*}$$ (24)

Proposition 2: By comparing and analyzing the changes of green level contribution, optimal profit and overall effect of supply chain of pharmaceutical manufacturers under single subsidy strategy and anarchy subsidy strategy, we can get the results as follows: $e_{r}^{*}-e_{n}^{*}≻0、z_{r}^{*}-z_{n}^{*}≻0$ (When the 4*βη*_*L*_−*γ*^2^≻0)、 $\pi_{r}^{L*}-\pi_{L}^{*}≻0、\pi_{r}^{R*}-\pi_{R}^{*}≻0$ (When the $\gamma^{4}(s-1)\eta_{R}^{2}+4\eta_{L}^{2}(\gamma^{2}-2\beta \eta_{R})\left[\gamma^{2}+2\beta (s-1)\eta_{R}\right]≻0$).

Proof: $e_{r}^{*}-e_{n}^{*}=\frac{(\alpha -c\beta )\gamma (s-1)\eta_{R}}{-\gamma^{2}(s-1)\eta_{R}+2\eta_{L}\left[\gamma^{2}+2\beta (s-1)\eta_{R}\right]}-\frac{\gamma \eta_{R}(\alpha -c\beta )}{-2\eta_{L}(\gamma^{2}-2\beta \eta_{R})-\gamma^{2}\eta_{R}}=\frac{2s\gamma^{3}\eta_{R}\eta_{L}(\alpha -c\beta )}{\left\{-\gamma^{2}(s-1)\eta_{R}+2\eta_{L}\left[\gamma^{2}+2\beta (s-1)\eta_{R}\right]\right\}\left\{2\eta_{L}(\gamma^{2}-2\beta \eta_{R})+\gamma^{2}\eta_{R}\right\}}$ Because $\beta^{2}(s-1)\eta_{R}\left\{-\gamma^{2}(s-1)\eta_{R}+2\eta_{L}\left[\gamma^{2}+2\beta (s-1)\eta_{R}\right]\right\}≻0,‐\gamma^{2}-2\beta (s-1)\eta_{R}≻0,e_{r}^{*}≻0,e_{n}^{*}≻0,$ so $e_{r}^{*}-e_{n}^{*}≻0$. In the same way but card $\pi_{r}^{L*}-\pi_{L}^{*}≻0$.

$z_{r}^{*}-z_{n}^{*}=\frac{(c\beta -\alpha )\gamma \eta_{L}}{-\gamma^{2}(s-1)\eta_{R}+2\eta_{L}\left[\gamma^{2}+2\beta (s-1)\eta_{R}\right]}-\frac{\gamma \eta_{L}(c\beta -\alpha )}{2\eta_{L}(\gamma^{2}-2\beta \eta_{R})+\gamma^{2}\eta_{R}}=\frac{\left(\alpha -c\beta )\gamma s\eta_{L}\eta_{R}(-\gamma^{2}+4\beta \eta_{L}\right)}{\left\{-\gamma^{2}(s-1)\eta_{R}+2\eta_{L}\left[\gamma^{2}+2\beta (s-1)\eta_{R}\right]\right\}\left\{2\eta_{L}(\gamma^{2}-2\beta \eta_{R})+\gamma^{2}\eta_{R}\right\}}$ Because $\beta^{2}(s-1)\eta_{R}\left\{-\gamma^{2}(s-1)\eta_{R}+2\eta_{L}\left[\gamma^{2}+2\beta (s-1)\eta_{R}\right]\right\}≻0,z_{r}^{*}≻0,z_{n}^{*}≻0,$ so [*γ*^2^*η*_*R*_+2*η*_*L*_(*γ*^2^−2*βη*_*R*_)]≺0, So whether $z_{r}^{*}-z_{n}^{*}$ is greater than 0 depends on 4*βη*_*L*_−*γ*^2^,when 4*βη*_*L*_−*γ*^2^≻0, $z_{r}^{*}-z_{n}^{*}≻0$; And vice, $z_{r}^{*}-z_{n}^{*}\leq 0$. In the same way but card, when $\gamma^{4}(s-1)\eta_{R}^{2}+4\eta_{L}^{2}(\gamma^{2}-2\beta \eta_{R})\left[\gamma^{2}+2\beta (s-1)\eta_{R}\right]≻0$, $\pi_{r}^{R*}-\pi_{R}^{*}≻0$, and vice, $\pi_{r}^{R*}-\pi_{R}^{*}\leq 0$.

Result analysis of Proposition 2: When the government only subsidizes pharmaceutical manufacturer, the contribution of pharmaceutical logistics provider to the green level of logistics is greater than that without subsidies. At the same time, the optimal profit of medical logistics companies is also greater than that under the condition of no subsidies. This indicates that the single subsidy strategy of pharmaceutical manufacturer also has a certain incentive effect on the logistics companies and has achieved good results. However, for manufacturer, the contribution of logistics green level under this strategy is greater than that under the anarchy subsidy strategy only when 4*βη*_*L*_−*γ*^2^≻0 is met. Similarly, the optimal profit is greater than that under the anarchy subsidy strategy only when $\gamma^{4}(s-1)\eta_{R}^{2}+4\eta_{L}^{2}(\gamma^{2}-2\beta \eta_{R})\left[\gamma^{2}+2\beta (s-1)\eta_{R}\right]≻0$ is satisfied. To sum up, when the government only provides green subsidies to manufacturer, the promotion effect of green logistics is greater than that of anarchy subsidy strategy, but the effect of subsidies is limited. Among them, the strategy has an obvious positive effect on the green logistics behavior of pharmaceutical logistics provider, but has a conditional limit on the incentive effect of the subsidy object itself. On this basis, under these circumstances, pharmaceutical logistics companies tend to accept this kind of subsidy arrangement. In order to maximize their own interests, manufacturers tend not to accept this situation. Because, in the process of logistics green logistics business always assume the main task, play a more critical role. Under this subsidy strategy, because only manufacturers get the subsidy, at this time, the logistics companies are more dependent on transferring the task of logistics greening to the manufacturers, which makes the manufacturers get the government subsidy, but need to pay more green input, leading to their profits are not necessarily increased. At the same time, manufacturers will have the conditions to improve their contribution to the green logistics. In such cases, pharmaceutical manufacturer will be cautious about accepting the policy.

### Coordinate subsidy strategy

In practice, the government's single subsidy strategy often fails to achieve the desired effect. Because pharmaceutical logistics provider plays a major role in green logistics transformation, they receive more direct subsidies, and manufacturer's contributions are often ignored. However, as manufacturer in the green packaging and green logistics promotion of the positive role gradually prominent, the government has also paid more and more attention to it. On this basis, in order to promote the greening of pharmaceutical logistics, the government formulates a coordinated subsidy strategy according to the actual situation, and subsidizes both pharmaceutical logistics provider and manufacturer. Under the internal coordination subsidy strategy, the profit functions of medical logistics provider and manufacturer are as follows:

$$\pi_{x}^{L}=(v_{x}-c)(\alpha -\beta p_{x}+\gamma e_{x}+\gamma z_{x})-\eta_{L}z_{x}^{2}/2+s\eta_{L}z_{x}^{2}/2$$ (25)

$$\pi_{x}^{R}=(p_{x}-v_{x})(\alpha -\beta p_{x}+\gamma e_{x}+\gamma z_{x})-\eta_{R}z_{x}^{2}/2+s\eta_{R}z_{x}^{2}/2$$ (26)

The total profit function of green supply chain is as follows:

$$\pi_{x}^{B}=(p_{x}-c)(\alpha -\beta p_{x}+\gamma e_{x}+\gamma z_{x})-\eta_{L}z_{x}^{2}/2-\eta_{R}z_{x}^{2}/2+s\eta_{L}z_{x}^{2}/2+s\eta_{R}z_{x}^{2}/2$$ (27)

And similarly, we're going to do it by backward induction, when −*γ*^2^−2*β*(*s*−1)*η*_*R*_≻0, {*γ*^2^*η*_*R*_+2*η*_*L*_[*γ*^2^+2*β*(*s*−1)*η*_*R*_]}≺0, the result is as follows:

$$\{\begin{array}{c} v_{x}^{*}=\frac{c\beta \gamma^{2}\eta_{R}+(\alpha +c\beta )\eta_{L}\left[\gamma^{2}+2\beta (s-1)\eta_{R}\right]}{\beta \left\{\gamma^{2}\eta_{R}+2\eta_{L}\left[\gamma^{2}+2\beta (s-1)\eta_{R}\right]\right\}}\\ e_{x}^{*}=\frac{(\alpha -c\beta )\gamma \eta_{R}}{\gamma^{2}\eta_{R}+2\eta_{L}\left[\gamma^{2}+2\beta (s-1)\eta_{R}\right]} \end{array}$$ (28)

$$\{\begin{array}{c} p_{x}^{*}=\frac{c\beta \gamma^{2}\eta_{R}+\eta_{L}\left[(\alpha +c\beta )\gamma^{2}+\beta (3\alpha +c\beta )(s-1)\eta_{R}\right]}{\beta \left\{\gamma^{2}\eta_{R}+2\eta_{L}\left[\gamma^{2}+2\beta (s-1)\eta_{R}\right]\right\}}\\ z_{x}^{*}=\frac{(-\alpha +c\beta )\gamma \eta_{R}}{\gamma^{2}\eta_{R}+2\eta_{L}\left[\gamma^{2}+2\beta (s-1)\eta_{R}\right]} \end{array}$$ (29)

Eq (28) and Eq (29) are substituted into Eq (25) and Eq (26) respectively to obtain the optimal profit:

$$\{\begin{array}{c} \pi_{x}^{L*}=\frac{{\left(\alpha -c\beta \right)}^{2}(s-1)\eta_{R}\eta_{L}}{2\gamma^{2}\eta_{R}+4\eta_{L}\left[\gamma^{2}+2\beta (s-1)\eta_{R}\right]}\\ \pi_{x}^{R*}=\frac{{\left(\alpha -c\beta \right)}^{2}(s-1)\eta_{L}^{2}\eta_{R}\left[\gamma^{2}+2\beta (s-1)\eta_{R}\right]}{2{\left\{\gamma^{2}\eta_{R}+2\eta_{L}\left[\gamma^{2}+2\beta (s-1)\eta_{R}\right]\right\}}^{2}} \end{array}$$ (30)

At this time, the optimal profit of the supply chain is as follows:

$$\pi_{x}^{B*}=\pi_{x}^{L*}+\pi_{x}^{R*}$$ (31)

Proposition 3: By comparing and analyzing the contribution of each participant to the green level of logistics under the subsidy strategy and the anarchy subsidy strategy, the change of the optimal profit is as follows:

$e_{x}^{*}-e_{n}^{*}≻0,\;z_{x}^{*}-z_{n}^{*}≻0,\pi_{x}^{L*}-\pi_{L}^{*}≻0,\pi_{x}^{R*}-\pi_{R}^{*}≻0$ ((*γ*^2^−2*βη*_*R*_){*γ*^2^*η*_*R*_+2*η*_*L*_[*γ*^2^+2*β*(*s*−1)*η*_*R*_]}^2^≻(1−*s*)[*γ*^2^+2*β*(*s*−1)*η*_*R*_][*γ*^2^*η*_*R*_+2*η*_*L*_(*γ*^2^−2*βη*_*R*_)]^2^)Proof: $\pi_{x}^{R*}-\pi_{R}^{*}=\frac{{\left(\alpha -c\beta \right)}^{2}(s-1)\eta_{L}^{2}\eta_{R}\left[\gamma^{2}+2\beta (s-1)\eta_{R}\right]}{2{\left\{\gamma^{2}\eta_{R}+2\eta_{L}\left[\gamma^{2}+2\beta (s-1)\eta_{R}\right]\right\}}^{2}}-\frac{{\left(\alpha -c\beta \right)}^{2}\eta_{L}^{2}\eta_{R}(2\beta \eta_{R}-\gamma^{2})}{2{\left[\gamma^{2}\eta_{R}+2\eta_{L}(\gamma^{2}-2\beta \eta_{R})\right]}^{2}}=\frac{{\left(\alpha -c\beta \right)}^{2}\eta_{L}^{2}\eta_{R}\left\{(\gamma^{2}-2\beta \eta_{R}){\left\{\gamma^{2}\eta_{R}+2\eta_{L}\left[\gamma^{2}+2\beta (s-1)\eta_{R}\right]\right\}}^{2}-(1-s)\left[\gamma^{2}+2\beta (s-1)\eta_{R}\right]{\left[\gamma^{2}\eta_{R}+2\eta_{L}(\gamma^{2}-2\beta \eta_{R})\right]}^{2}\right\}}{2{\left\{\gamma^{2}\eta_{R}+2\eta_{L}\left[\gamma^{2}+2\beta (s-1)\eta_{R}\right]\right\}}^{2}{\left[\gamma^{2}\eta_{R}+2\eta_{L}(\gamma^{2}-2\beta \eta_{R})\right]}^{2}}$ From the foregoing we know that: −*γ*^2^−2*β*(*s*−1)*η*_*R*_≻0,[*γ*^2^*η*_*R*_+2*η*_*L*_(*γ*^2^+2*β*(*s*−1)*η*_*R*_)]≺0,2*βη*_*R*_−*γ*^2^≻0, so when (*γ*^2^−2*βη*_*R*_){*γ*^2^*η*_*R*_+2*η*_*L*_[*γ*^2^+2*β*(*s*−1)*η*_*R*_]}^2^≻(1−*s*)[*γ*^2^+2*β*(*s*−1)*η*_*R*_][*γ*^2^*η*_*R*_+2*η*_*L*_(*γ*^2^−2*βη*_*R*_)]^2^, $\pi_{x}^{R*}-\pi_{R}^{*}≻0$. Same as proposition 2, proposition 3 can be proved $e_{x}^{*}-e_{n}^{*}≻0,z_{x}^{*}-z_{n}^{*}≻0,\pi_{x}^{L*}-\pi_{L}^{*}≻0$.

Result analysis of Proposition 3: Compared with anarchy subsidy strategy, under the coordinated subsidy strategy of the government, the contribution of pharmaceutical logistics provider to the green level of logistics and their corresponding optimal profits are greater than that of manufacturer. And the optimal profit of the manufacturer is greater than the optimal profit without subsidies only at (*γ*^2^−2*βη*_*R*_){*γ*^2^*η*_*R*_+2*η*_*L*_[*γ*^2^+2*β*(*s*−1)*η*_*R*_]}^2^≻(1−*s*)[*γ*^2^+2*β*(*s*−1)*η*_*R*_][*γ*^2^*η*_*R*_+2*η*_*L*_(*γ*^2^−2*βη*_*R*_)]^2^. This indicates that the government coordination subsidy strategy has a relatively obvious positive incentive effect on the green logistics transformation behavior of pharmaceutical logistics provider and a restrictive incentive positive effect on manufacturer. Since the contribution of the number of participants in this strategy is greater than that of the anarchy subsidy strategy, this subsidy strategy can effectively strengthen the motivation of pharmaceutical logistics provider and manufacturer to implement green logistics behavior, but it has certain restrictions on the improvement of the overall green level of logistics. On this basis, pharmaceutical logistics companies are more willing to accept this subsidy, while pharmaceutical manufacturer tends to reject this subsidy strategy with the goal of profit maximization. This is not consistent with the conventional wisdom. Because under the subsidy policy, both parties are subsidized, each party is willing to increase their contribution to the green level of pharmaceutical logistics. However, due to the strong position of logistics providers in the supply chain, manufacturers will undertake more green tasks, such as increasing the green requirements for the packaging of the goods themselves, and reducing the transportation packaging requirements of logistics companies The cost of flow business will be reduced while the cost of manufacturer will be further increased, which will hit the enthusiasm of pharmaceutical manufacturers.

## Conclusion

This paper mainly focuses on the green supply chain composed of pharmaceutical logistics provider and manufacturer, considering the preference of green logistics, and studies the influence of different government subsidy strategies on the green process of pharmaceutical logistics. First, the paper studies the behavior selection and optimal decision of each participant in the green supply chain without government subsidy strategy. Furthermore, it analyzes the optimal choice of each participant under the single subsidy strategy of pharmaceutical logistics providers, the single subsidy strategy of pharmaceutical manufacturers and the coordinated subsidy strategy. On this basis, the paper compared and analyzed the influence of three different subsidy strategies on the optimal profit of supply chain members and the contribution of logistics green level, and then analyzed the influence of different government subsidy strategies on the green transformation of pharmaceutical logistics. At the same time, the paper analyzes the behavioral choices of each member of the supply chain based on the influence of different subsidy strategies on their own optimal profits.

The results show that: compared with the case of no subsidy, the three different subsidies have different degrees of incentive effect on the green behavior of pharmaceutical manufacturers and logistics providers. The government and its participants can make behavioral decisions according to the research results. For the government, the implementation of the single subsidy strategy for pharmaceutical logistics providers will encourage pharmaceutical logistics providers and manufacturers to improve their contribution to the green level of logistics; the implementation of single subsidy strategy for pharmaceutical manufacturers will encourage pharmaceutical retailers to improve their contribution to the green level of logistics, but the incentive effect on pharmaceutical manufacturers is limited; the implementation of coordinated subsidy strategy will also encourage pharmaceutical logistics providers and manufacturers to improve their contribution to the green level of logistics. For each participant in the supply chain, based on the goal of maximizing their own profits, under the single subsidy strategy of pharmaceutical logistics providers, both pharmaceutical logistics providers and retailers tend to accept the strategy; under the single subsidy strategy of pharmaceutical manufacturers, pharmaceutical manufacturers tend to accept the strategy, while pharmaceutical manufacturers tend to reject the strategy; under the coordinated subsidy strategy, pharmaceutical logistics providers tend to accept the strategy, while pharmaceutical manufacturers tend to reject it. Compared with the results of the above strategy analysis, the single subsidy strategy adopted by the government has the most obvious promotion effect on the green transformation of pharmaceutical logistics. This paper only considers the game model between a single pharmaceutical logistics provider and a single manufacturer. In the future, competition mechanism can be introduced to consider a single logistics provider and multiple manufacturers or multiple logistics provider and a single manufacturer to build a supply chain game model. At the same time, the social welfare evaluation mechanism can be introduced to enrich the measurement indicators.

## Supporting information

S1 Table Variable parameter symbol description. A list of notations introduced so far is provided in S1 Table.
(DOCX)
