# Supplementary material for: Research on green decision making of pharmaceutical logistics considering government subsidy strategy
Source: PLoS One. 2020 Oct 30;15(10):e0241400. doi: 10.1371/journal.pone.0241400 (PMC7598517; doi:10.1371/journal.pone.0241400)
Supplement: S1 Table — A list of notations introduced so far is provided in S1 Table. (DOCX) [file pone.0241400.s001.docx]

**S1 Table. Variable Parameter Symbol Description.**

| Symbol | Definition |
| --- | --- |
| c | Traditional logistics cost |
| v | Logistics provider unit logistics price |
| p | Unit logistics fees charged by a manufacturer to a retailer |
| s | Government green logistics subsidy coefficient |
| z | Manufacturer logistics green level contribution |
| e | Contribution of green level of logistics by logistics companies |
| γ | Retailers prefer green logistics |
| $\eta_{L}$ | Logistics provider green input cost coefficient |
| $\eta_{R}$ | Manufacturer green input cost coefficient |
